# Lord Lister: His Work, His Teaching, and Benefits to Mankind

**Published:** 1918-01-26

**Authors:** 


### LORD LISTER.

### His Work, His Teaching, and Benefits to Mankind.

DURING the three years and a half that the war has lasted more than four million casualties have been officially recorded among the Germans alone, and the recorded casualties are far fewer than the casualties that have been suffered. It is computed that at least a million and three-quarters of Germans have been killed. The Austro-Hungarian forces are in number about 80 per cent. of the German, and they have suffered in proportion to their number more severely than the Germans. The number of killed in the two armies cannot amount to fewer than three millions. Their adversaries have lost in killed, in the aggregate, including the Russians, quite as many. Add to these the slaughter of the Belgians, the Serbians, the Armenians, and the other races subject to the Turk, and the total mortality caused by the Great War during the three and a half years that it has been raging cannot be less than seven millions of able-bodied men. If we add to these the deaths directly owing to the war, but not inflicted by shell, bullet, or bayonet, deaths from starvation, deaths from cold, deaths by drowning, deaths from war-diseases, deaths from the effect of combined privations, cold, hunger, and misery co-operating, and it will be no exaggeration to say that the action of the Kaiser and his satellites in designing and producing the war has brought about the deaths of not less than eight millions of human beings. The sum total is sufficiently appalling; but it is certain that during the same period of three and a half years since the commencement of the war twice as many lives have been saved by the practices initiated by Lister.

The wounded in war have been more than twice as many as the killed: they have been three or four times as many; and if we may go by what has happened in previous wars, we may say with certainty that but for Listerism by far the greater number of the wounded would have died of their wounds. And during the war the civil surgeons have not been idle: even in the belligerent countries operations among civilian men and women have still been performed in great numbers. Hospitals have still been full. Surgeons have still been fully employed. Women have still given birth to children, even in belligerent countries; and all the world over life has been going on on very much the old lines; and in every country with any pretension to civilisation Listerism is an established practice. Among the two hundred millions in North and South America who were not until recently concerned in the war life has been going on as usual; life has been subject to accidents and diseases, and wherever accidents and diseases are treated in civilised countries Listerism has been put in force, and Listerism has saved lives. Precisely how many lives it has saved during the last three and a half years we have no means of knowing; but we can be quite sure that it is not less than the number that the war has destroyed.

Since the nameless and forgotten benefactor who invented the plough, Lister was the greatest benefactor of the human race that the human race has ever known. In classical times, temples would have been erected in his honour, and priesthoods consecrated to his worship. Statues of him would have been in every household; his praise would have been on every lip; days of thanksgiving to him would have been set apart in the calendar; the world would have rung with praise, honour, and thankfulness to Lister; and what did we do for him? We are the children of them that stoned the prophet of cleanliness and purity. When he was indifferent and could not enjoy it; when he was solitary and could not impart it; when he was known and did not want it; then he was elevated—elevated!—to the lowest rank in the peerage; then he was graciously permitted to sit cheek by jowl with wealthy brewers, with self-seeking purveyors of false news, with thick-headed country squires, and with contributors on the grand scale to party funds, used for the purpose of foisting, among others, a German spy into the English House of Commons. The eightieth birthday of the greatest benefactor the human race has ever known was honoured by many prominent men in many parts of the earth. In his own country it passed almost unnoticed, quite unnoticed by the mass of his fellow-countrymen, and without even a message of congratulation from the King, the official head and representative of the nation that was honoured by having produced him, and doubly honoured by having witnessed the success which the nation of his birth so tardily and reluctantly acknowledged. So

> Nations slowly wise and meanly just
> To buried merit raise the tardy bust.

In no country have so many great and momentous discoveries and inventions been made as in this: in no country have their inventions and discoveries been as much neglected and ridiculed as in this: in no country has acknowledgment been so tardy, grudging, and unwilling: in no country has the inventor or discoverer been so often left to end his days in want. Fortunately for himself, and most fortunately for the world at large, Lister was raised by parental care above the reach of want, and it is a sufficient reply to the silly clamour for the "conscription of wealth" that if it were in force, not only would one of the greatest motives to human activity be abolished, but also such momentous discoveries as those of Lister would become so difficult as to be almost impossible.

It is six years since Lister died, at a ripe age and full of honours, and now at length a full and authoritative life of him has been published by Messrs. Macmillan. It is written with pious care and with great biographical skill and literary ability by Sir Rickman Godlee, Lister's nephew, his assistant for many years in professional work, and his life-long pupil and friend. Such a book requires, in

**LORD LISTER.**—(*continued*).

a weekly review like THE HOSPITAL, which is read not only by the medical profession but by many outside the professional ranks, something more than an ordinary review. It is not merely the biography of a great man, it is the history of a great discovery. It is more. It is the history of a revolution, of a revolution that, in its consequences to humanity at large, does not suffer by comparison with the great French Revolution of more than a hundred years ago. In the clangour and din of a great war the voice of history is overpowered, and the lay newspapers have reviewed this Life of Lord Lister as if it were no more than the life of an undistinguished bishop, or a second or third rank politician. We propose to treat it more in accordance with its importance.

(*To be continued.*)

